# The causal interaction in human basal ganglia

**DOI:** 10.1038/s41598-021-92490-8

**Published:** 2021-06-21

**Authors:** Clara Rodriguez-Sabate, Albano Gonzalez, Juan Carlos Perez-Darias, Ingrid Morales, Manuel Rodriguez

**Affiliations:** 1 Laboratory of Neurobiology and Experimental Neurology, Department of Basic Medical Sciences, Faculty of Medicine, University of La Laguna, Tenerife, Canary Islands Spain; 2 Center for Networked Biomedical Research in Neurodegenerative Diseases (CIBERNED), Madrid, Spain; 3 Department of Psychiatry, Getafe University Hospital, Madrid, Spain; 4 Department of Physic, University of La Laguna, Tenerife, Canary Islands Spain

**Keywords:** Neuroscience, Physiology, Systems biology

## Abstract

The experimental study of the human brain has important restrictions, particularly in the case of basal ganglia, subcortical centers whose activity can be recorded with fMRI methods but cannot be directly modified. Similar restrictions occur in other complex systems such as those studied by Earth system science. The present work studied the cause/effect relationships between human basal ganglia with recently introduced methods to study climate dynamics. Data showed an exhaustive (identifying basal ganglia interactions regardless of their linear, non-linear or complex nature) and selective (avoiding spurious relationships) view of basal ganglia activity, showing a fast functional reconfiguration of their main centers during the execution of voluntary motor tasks. The methodology used here offers a novel view of the human basal ganglia which expands the perspective provided by the classical basal ganglia model and may help to understand BG activity under normal and pathological conditions.

## Introduction

The experimental study of the human brain presents important restrictions, particularly in the case of the deep brain nuclei. The basal ganglia (BG) are located under the brain cortex and cannot be directly manipulated, but their activity can be recorded with non-invasive methods. Magnetic resonance imaging (MRI) can provide information about the functional activity (functional MRI; fMRI), and functional connectivity (functional connectivity MRI; fcMRI) of the BG of subjects who are performing particular tasks^1–5^. These methods use the dynamics of the blood oxygenation level dependent (BOLD) signal, to quantify the vasodilatation (relative concentration of oxy- and deoxy-hemoglobin) induced by brain activity. The increase in the BOLD signal is normally used to identify the brain centers involved in particular tasks, and the time-relationship of the BOLD signal fluctuation of two centers (time-series) is often used to analyze the functional interaction of these centers. Unfortunately, only statistical dependencies can be evaluated from these observational data, and the cause/effect relationship necessary to develop robust models of the brain activity may not be properly established. Similar handicaps are present in other complex systems such as those studied by the Earth system science, and where the variables involved in their functional dynamics can be monitored but not experimentally manipulated. Thus, there is a growing interest to develop mathematical frameworks suitable for studying the cause/effect relationship of these complex systems by analyzing the time-series generated by the activity of their components. These methods, which began with the seminal studies of Wiener and Granger (1950s–1960s), have evolved rapidly^6–13^, and are being used successfully for the study of several climate signals and the dependence between them^8^. Different European groups (e.g., the Aerospace Center in Germany and the Grantham Institute of the Imperial College in London) have recently introduced new methods which facilitate the study of the cause/effect relationships involved in climate dynamics^8,10,11^. Unlike the study of correlations between pairs of time series, the methods for reconstructing causal networks allow us to distinguish between direct and indirect dependencies, in addition to detecting common drivers in a set of time series. These methods were used here to study the causality relationships between BG nuclei.

During the 1980s and 1990s, BG were grouped in four cortico-subcortical closed-loop circuits by a model which was later used to explain the role of each nucleus in the physiology and physiopathology of BG. This model, which is widely used today, arranges BG in a serial succession of centers which process the information received from the brain cortex, and return the processed information to its cortical origin (cortico-subcortical loop). One of these closed-loop BG networks is the BG motor circuit (BGmC), which receives the motor information provided by the primary motor cortex (M1), successively processes this information in the putamen (Put), external globus pallidum (GPe), subthalamic nucleus (STN), internal globus pallidum (GPi) and substantia nigra (SN), and finally returns the processed information to the M1 through the anterior thalamus (motor thalamus; Tal). Thus, the M1 is considered as both the input and output of the BGmC, and is also the cortical center which finally controls the neurons that directly modulate muscle activity. The BGmC is composed of three parallel closed-loop networks, the direct (M1 → Put → SNr/GPi → Tal → M1), the indirect (M1 → Put → GPe → STN → GPi/SNr → Tal → M1) and the hyperdirect (M1 → STN → SNr/GPi → Tal → M1) cortico-subcortical closed-loops. In the classical BG model, the three loops compete for the functional control of the M1 activity^14–16^. Although this model has been successfully applied to explain some motor symptoms of BG disorders^16–24^, other symptoms remain unexplained^18,19,25^, and there is a growing interest in updating this BG model^26–32^. The classical BG model uses the excitatory/inhibitory interactions of BG neurons recorded in experimentation animals to estimate the cause/effect relationship of BG centers and the overall dynamics of the human BGmC. Different methods have recently been introduced to study the functional relationships between human BG^31–33^ but the cause/effect interaction of these centers has never been directly tested in humans. In addition, a number of subcortical pathways that may be critical for understanding the global dynamics of BG are often not included in the classical BG model^34^. The present work uses the causation analysis developed to study the climate and other complex systems that cannot be experimentally manipulated to study the functional relationships of the human BGmC with fcMRI recordings.

## Results

Three complementary statistical methods were used here to identify the functional interactions of BG nuclei. The first one is based on classical statistics and provides a robust theoretical background. It assumes linear relationships between variables, testing the conditional independence through the corresponding partial correlation (PC). The second one uses a non-parametric method based on gaussian process regression and a distance correlation (GPDC)^35^ to test the dependence, allowing the detection of non-linear dependencies. The last one is the conditional mutual information test based on nearest-neighbor (CMIknn) estimator^36^. It is the most general dependency measure, and makes no assumptions about the parametric form of the dependencies by directly estimating the underlying joint density. The non-parametric and model-free methods allow the detection of non-linear relationships in complex systems, but they are based on weaker theoretical results. These methods were used to identify instantaneous dependencies and delayed causations. The instantaneous dependency indicates that two nuclei present functional relationships but, because it occurs within the same time-window of the fcMRI methods (1.6 s), the nuclei which activate this relationship cannot be identified. The delayed causation shows a slow functional interaction between two nuclei (one nucleus is activated at least 1.6 s before the other nucleus). The nucleus activated first is considered as the cause of this interaction.

**Figure 1:**
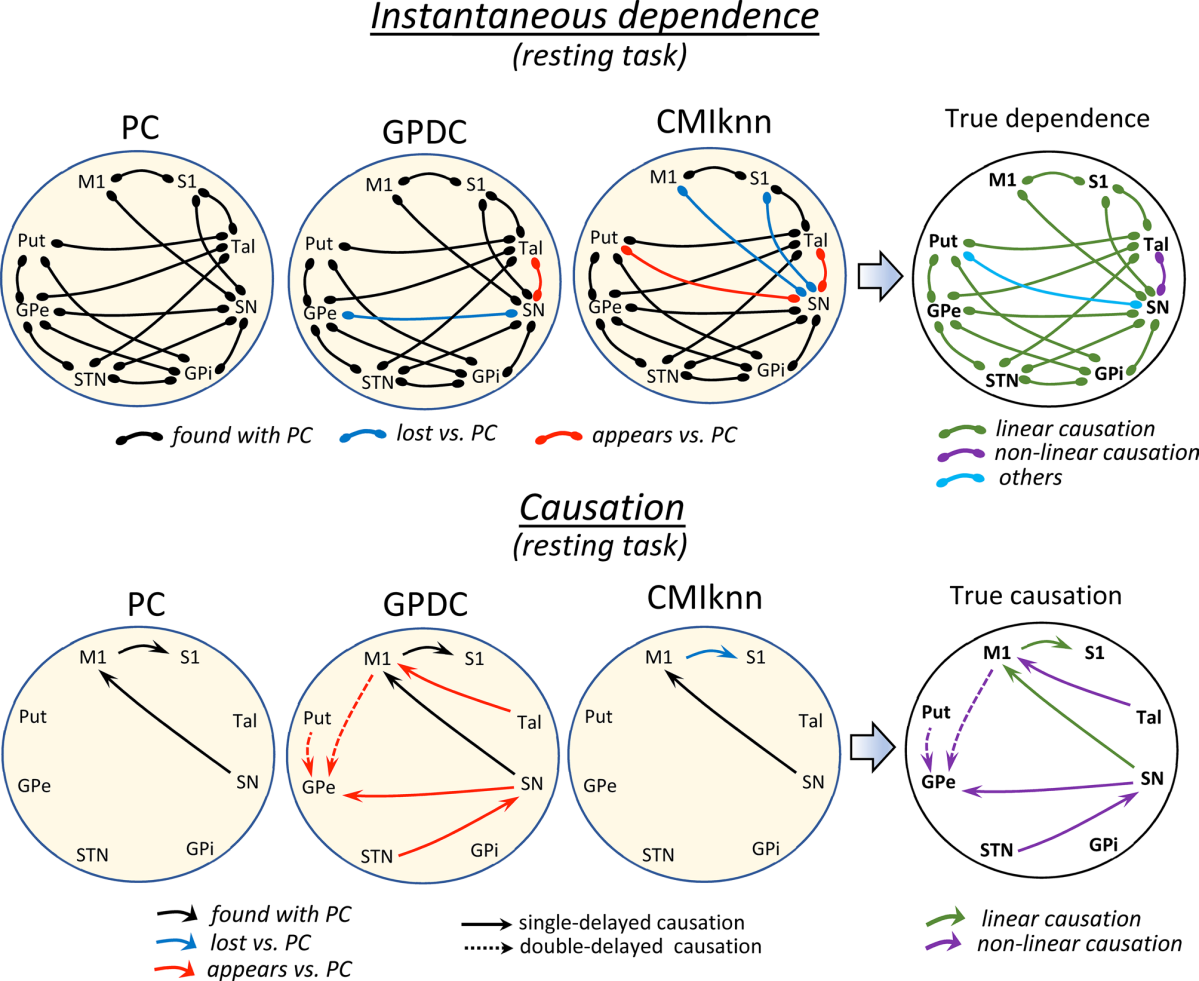
Instantaneous dependence vs. delayed causation during the resting task. Connections between centers show dependence or causation relationships with statistical value (*p* < 0.01). A summary of the main dependence relationships is shown at the top-right (true dependence), and a summary of the main causation relationships is shown at the bottom -right (true causation). PC: partial correlation, GPDC: Gaussian process regression and distance correlation, CMIknn: conditional mutual information test (based on nearest-neighbor). M1: primary motor cortex, S1: primary somato-sensory cortex, Put: putamen, GPe: external globus pallidum, STN: subthalamic nucleus, GPi: internal globus pallidum, SN: substantia nigra, Tal: motor thalamus.

Figure 1 shows the instantaneous dependency (top) and delayed causation (bottom) found with PC, GPDC and CMIknn methods during the resting intervals. PC showed 15 instantaneous and 2 delayed dependencies which were also found with the GPDC methods. The only exception was the GPe-SN instant dependency that was observed with PC but not with GPDC (although GPDC identifies the linear relationships found by PC, it has less sensitivity than PC for this type of causation). In addition to the linear dependencies found by PC, GPDC showed 1 instantaneous and 5 delayed (3 single-delayed and 2 double-delayed) non-linear dependencies. CMIknn can detect all types of dependencies but it is much less sensitive than PC for linear dependencies and than GPDC for non-linear dependencies. Nonetheless, MCIknn identified most of the linear and non-linear instantaneous dependencies found by the other two methods, also showing an SN-Put instantaneous dependency not found by the other methods and whose characteristics are not presently identified. The low sensitivity of CMIknn was more clearly observed in the delayed causation where only the SN → M1 single-delayed linear causation was detected with this method. The right-side of Fig. 1 shows a summary of the main relationships (true dependencies), indicating their linear (green), non-linear (purple) or complex (cyan) nature. Taken together, present data show 17 instantaneous dependencies (15 linear, 1 non-linear and 1 complex) and 7 delayed causations (2 linear, 3 non-linear single-delayed and 2 non-linear double delayed).

**Figure 2:**
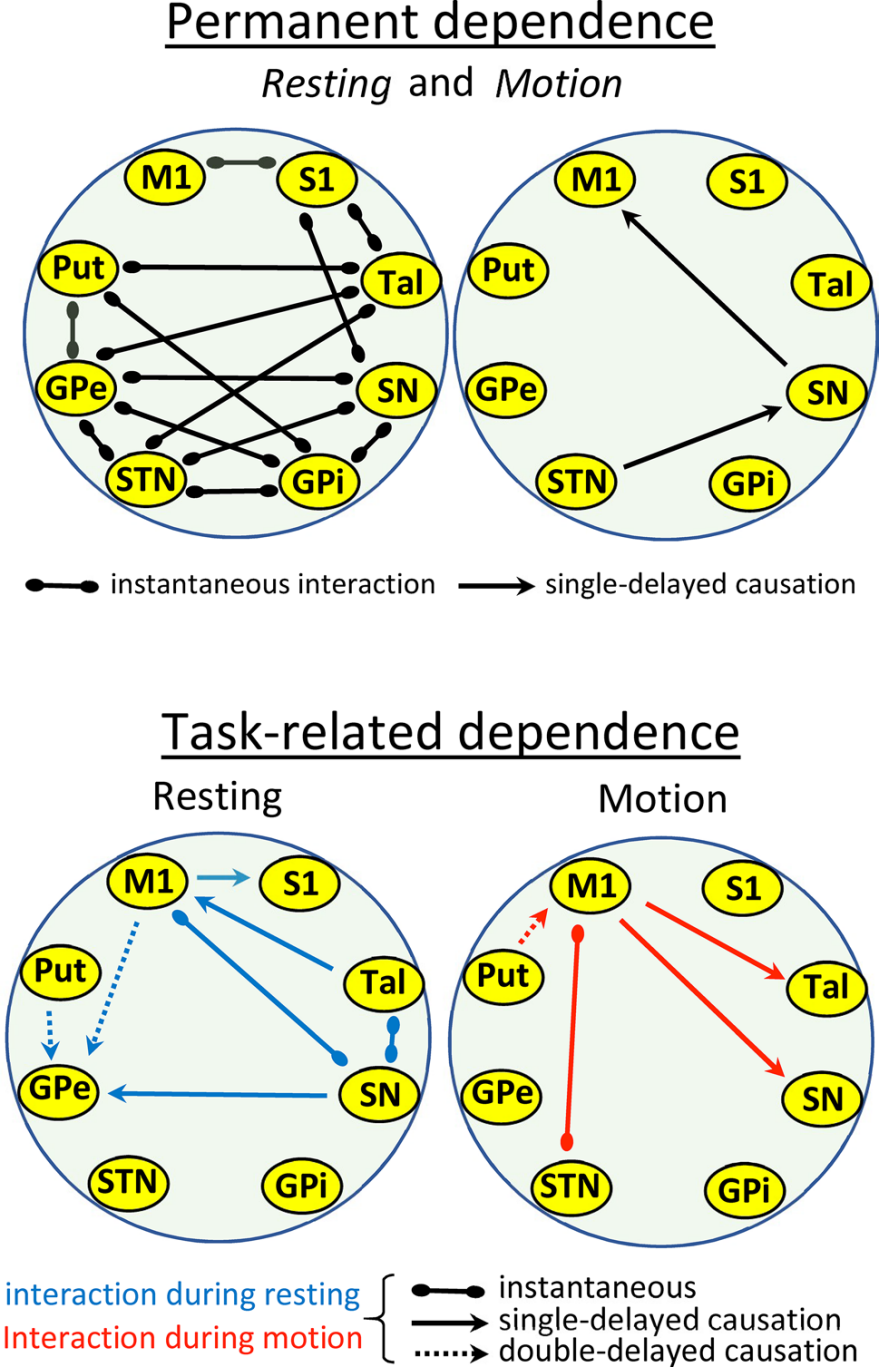
Permanent (resting + motion tasks) and task-dependent interactions. Connections between centers show relationships with statistical value (*p* < 0.01). M1: primary motor cortex, S1: primary somato-sensory cortex, Put: putamen, GPe: external globus pallidum, STN: subthalamic nucleus, GPi: internal globus pallidum, SN: substantia nigra, Tal: motor thalamus.

Figure 2 shows the task-dependent (resting-task in blue and motion-task in red) and task-independent (permanent dependencies found in both resting and motor tasks are shown in black) instantaneous and delayed dependencies. The statistical value of each functional interaction during the resting-task and motor-task is shown in Tables 1 and 2, respectively). Out of the 140 possible permanent dependencies (42 instantaneous+ 49 single-delayed+ 49 double-delayed causations), 30 showed a frequency higher than expected at random, with most of them being instantaneous dependencies (28 instantaneous+ 2 single-delayed dependencies). Most permanent instantaneous dependencies were produced between BG nuclei (11), and only 3 involved cortical areas (1 between M1 and S1 and 2 between BG and S1). Only 2 permanent delayed causations were found (STN → SN and SN → M1). Out of the 280 possible task-dependent dependencies (84 instantaneous+ 98 single-delayed+ 98 double-delayed causations), 11 showed a frequency higher than expected at random, with most of them being delayed causations (3 instantaneous+ 5 single-delayed+ 3 double -delayed causations). Most task-dependent dependencies involved the M1.

**Table 1:** Statistical values of each functional link during the resting-task computed with the partial correlation (PC), gaussian process regression and distance correlation (GPDC), and conditional mutual information test (CMIknn) methods.

Re sponse\causative	Method	M1	S1	Put	GPe	STN	GPi	SN	Tal
**Lag 0**									
M1	PC GPDC CMIknn		1 1 0.704					− 0.139 0.122	
S1	PC GPDC CMIknn	1 1 0.704						− 0.149 0.021	− 0.224 0.047 0.066
Put	PC GPDC CMIknn				0.457 0.205 0.169		0.322 0.124 0.129	0.073	0.542 0.205 0.223
GPe	PC GPDC CMIknn			0.457 0.205 0.169		0.123 0.078 0.102	0.452 0.120 0.162	0.212 0.073	0.432 0.089 0.149
STN	PC GPDC CMIknn				0.123 0.078 0.102		0.551 0.316 0.284	0.970 0.981 0.514	0.374 0.044 0.109
GPi	PC GPDC CMIknn			0.322 0.124 0.129	0.452 0.120 0.162	0.551 0.316 0.284		0.251 0.049 0.150	
SN	PC GPDC CMIknn	− 0.139 0.122	− 0.149 0.021	0.073	0.212 0.073	0.970 0.981 0.514	0.251 0.049 0.150		0.031 0.118
Tal	PC GPDC CMIknn		− 0.224 0.047 0.066	0.542 0.205 0.223	0.432 0.089 0.149	0.374 0.044 0.109		0.031 0.118	
**Lag 1**									
M1	PC GPDC CMIknn							0.257 0.056 0.097	0.014
S1	PC GPDC CMIknn	− 0.637 0.377							
Put	PC GPDC CMIknn								
GPe	PC GPDC CMIknn							0.013	
STN	PC GPDC CMIknn								
GPi	PC GPDC CMIknn								
SN	PC GPDC CMIknn					0.127			
Tal	PC GPDC CMIknn								
**Lag 2**									
M1	PC GPDC CMIknn								
S1	PC GPDC CMIknn								
Put	PC GPDC CMIknn								
GPe	PC GPDC CMIknn	0.012		0.090					
STN	PC GPDC CMIknn								
GPi	PC GPDC CMIknn								
SN	PC GPDC CMIknn								
Tal	PC GPDC CMIknn								

Columns correspond to causative nuclei and rows to response nuclei. Only those relationships with *p*-values < 0.01 are shown. All the statistical values in this table were normalized between 0 and 1, a procedure that facilitates the comparison of the results obtained with the three methods. *M1* primary motor cortex, *S1* primary somato-sensory cortex, *Put* putamen, *GPe* external globus pallidum, *STN* subthalamic nucleus, *GPi* internal globus pallidum, *SN* substantia nigra, *Tal* motor thalamus.

**Table 2:** Statistical values of each functional link during the motor-task computed with the partial correlation (PC), gaussian process regression and distance correlation (GPDC), and conditional mutual information test (CMIknn) methods.

Response\causative	Method	M1	S1	Put	GPe	STN	GPi	SN	Tal
**Lag 0**									
M1	PC GPDC CMIknn		0.909 0.983 0.561			− 0.149 0.032			
S1	PC GPDC CMIknn	0.909 0.983 0.561						− 0.243 0.027 0.088	− 0.162 0.044 0.112
Put	PC GPDC CMIknn				0.516 0.177 0.292		0.303 0.085 0.143		0.534 0.216 0.254
GPe	PC GPDC CMIknn			0.516 0.177 0.292		0.177 0.022 0.116	0.403 0.092 0.259	0.269 0.011 0.144	0.364 0.051 0.211
STN	PC GPDC CMIknn	− 0.149 0.032			0.177 0.022 0.116		0.520 0.252 0.282	1 1 0.725	0.359 0.077 0.289
GPi	PC GPDC CMIknn			0.303 0.085 0.143	0.403 0.092 0.259	0.520 0.252 0.282		0.277 0.016 0.189	
SN	PC GPDC CMIknn		− 0.243 0.027 0.088		0.269 0.011 0.144	1 1 0.725	0.277 0.016 0.189		
Tal	PC GPDC CMIknn		− 0.162 0.044 0.112	0.534 0.216 0.254	0.364 0.051 0.211	0.359 0.077 0.289			
**Lag 1**									
M1	PC GPDC CMIknn							0.226 0.036	
S1	PC GPDC CMIknn								
Put	PC GPDC CMIknn								
GPe	PC GPDC CMIknn								
STN	PC GPDC CMIknn								
GPi	PC GPDC CMIknn					0.012			
SN	PC GPDC CMIknn	0.158 0.110				0.186			0.120
Tal	PC GPDC CMIknn	0.014							
**Lag 2**									
M1	PC GPDC CMIknn			0.137 0.020 0.112					
S1	PC GPDC CMIknn								
Put	PC GPDC CMIknn								
GPe	PC GPDC CMIknn								0.027
STN	PC GPDC CMIknn								
GPi	PC GPDC CMIknn					0.014			
SN	PC GPDC CMIknn								
Tal	PC GPDC CMIknn								

Columns correspond to causative nuclei and rows to response nuclei. Only those relationships with *p*-values < 0.01 are shown. All the statistical values in this table were normalized between 0 and 1, a procedure that facilitates the comparison of the results obtained with the three methods. *M1* primary motor cortex, *S1* primary somato-sensory cortex, *Put* putamen, *GPe* external globus pallidum, *STN* subthalamic nucleus, *GPi* internal globus pallidum, *SN* substantia nigra, *Tal* motor thalamus.

**Figure 3:**
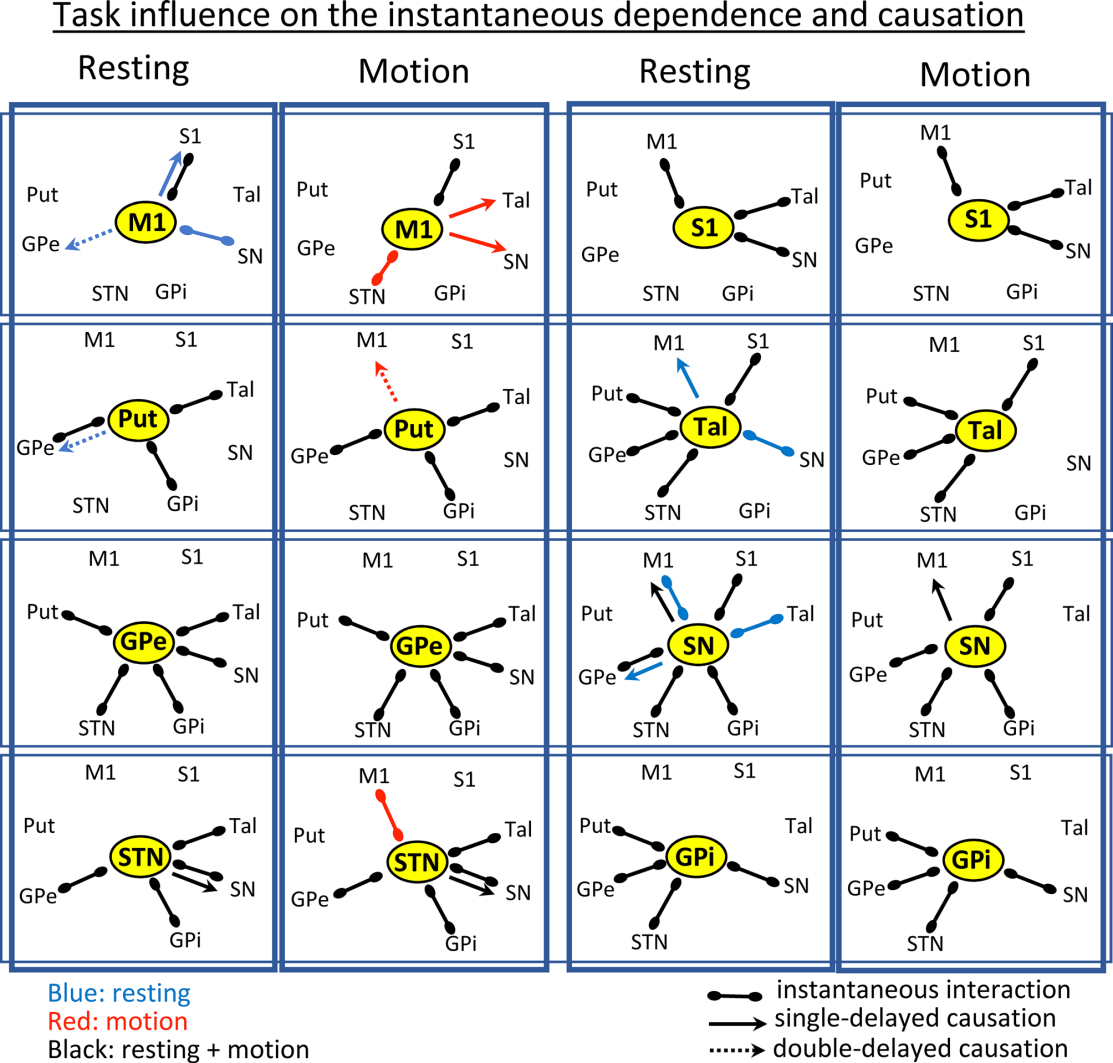
Influence of tasks on the functional interactions of BG. Connections between centers show relationships with statistical value (*p* < 0.01). Blue lines show the statistical interactions only observed during the resting task. Red lines show the statistical interactions only observed during the motor task. Black lines show the statistical interactions observed during both resting and motor tasks. M1: primary motor cortex, S1: primary somato-sensory cortex, Put: putamen, GPe: external globus pallidum, STN: subthalamic nucleus, GPi: internal globus pallidum, SN: substantia nigra, Tal: motor thalamus.

Figure 3 shows the relationships for each region of the BGmC. M1 and S1 showed a completely different set of relationships with the other centers of the BGmC. M1 showed an instantaneous and a delayed dependency with S1 during the resting-task (M1 working as causative nucleus). The brain area of the BGmC which most changed with the motor tasks was M1. Most M1 interactions with BG were delayed (6 delayed vs. 2 instantaneous) task-dependent (7 task-dependent vs. 1 permanent) dependencies. M1 worked as a causative center of the GPe activity during resting, and of the SN and Tal activity during the motion-task. In addition, M1 was a response center for the Tal activity during the resting-task. The instantaneous dependencies of M1 also changed with tasks, and were linked to SN during resting and with STN during motion. Thus, most M1-BG interactions were activated by particular motor tasks, with the SN → M1 being the only delayed causation observed during both the resting and motor tasks. S1 showed only three instantaneous relationships (with SN, Tal and M1) that did not change with the motor task.

Put showed instantaneous dependencies with GPe, GPi and Tal which did not change with the motor task. In addition, Put acted as a causative center of a double-delayed causation with both the GPe (during the resting-task) and M1 (during the motor-task). GPe showed an instantaneous connectivity with Put, STN, GPi, SN and Tal during the resting-task that did not change during the motor-task. STN showed an instantaneous interaction with GPe, GPi, SN and Tal during the resting-task which did not change during the motor task. STN acted as a single-delay causative center of the SN activity, a behavior that was similar during the resting and motor tasks. Similarly to that observed for the GPe, GPi showed an instantaneous dependency with different BG (Put, GPe, STN and SN) during resting that did not change with the motor-task. Thus, the main difference between GPe and GPi was the interaction with Tal, which was found for GPe but not for GPi. SN was the BG region with the highest number of interactions (8 different interactions). SN showed five permanent dependencies, which were instantaneous in four cases (GPe, STN, GPi and S1) and single-delayed in one case (M1). In addition, SN showed three task-dependent interactions (instantaneous with Tal and M1 and single-delayed with GPe) which were observed during the resting-interval and vanished during the motor-interval. Tal showed permanent instantaneous dependency with Put, GPe, STN and S1. In addition, Tal showed an instantaneous interaction with SN during the resting-task that vanished during the motor-task intervals, and also showed a single-delayed causation with M1 which was observed during the resting tasks.

## Discussion

The causation methods previously developed to study the evolution of atmospheric phenomena and other complex systems showed here an unprecedented vision of the intricate activity of the human BG. The combination of three dependent statistics procedures identified linear, non-linear and complex functional relationships between the main nuclei of the BGmC, also showing their functional connectivity with the primary motor cortex and primary somatosensory cortex. A portion of detected functional interactions (those performed in a time-window longer than the 1.6 s of the fMRI recording interval) showed causality relationships, thus identifying the causative and the response center. The rapid functional relationships (time-window less than 1.6 s) were also identified but, in this case, the causative center could not be recognized. Present methods showed an exhaustive (identifying all interactions regardless of their linear, non-linear or complex nature) and selective (avoiding the spurious relationship generated by the closed-loop arrangement of BG) view of the BGmC behavior. The functional interactions changed with the task, showing an unstable functional connectivity of BG that facilitates their fast reconfiguration during the execution of different motor tasks. The methodology used here offers a novel view of the human BG which expands the perspective provided by the classical BG models and may help to understand the BG activity under normal and pathological conditions.

### Methodological comments

The study of the internal dynamics of the human neural networks during the execution of particular motor task is presently a challenging goal for neuroscience, particularly when the nuclei involved in these networks are located deep in the brain and their experimental manipulation is not possible. This is the case of the BG, whose cause/effect relationships were studied here by analyzing BOLD signals recorded from the main BG with new methods which have recently been introduced to research causal relationships in complex systems where an experimental approach is not possible^8,10,11^. The causality which these methods provide is not exactly the same causality provided by the experimental studies. The “*experimental causality*” considers X to be the cause of Y only when the repeated manipulation of X has the same effect on Y, a manipulation which cannot generally be performed in human BG. What can be studied with present method is the “*statistical causality*”, the probability that the finding of X will be followed by the finding of Y. Different definitions have been proposed for statistical causality (bivariate Granger causality, bivariate transfer entropy, conditional mutual information, phase transfer entropy, etc..), with the time-sequence of events (X → Y) being the main characteristic to identify causes (previous events) and effects (subsequent events) in all these definitions. All the causality methods require a number of preconditions (stationarity, causal sufficiency, faithfulness..) that cannot always be verified (particularly in brain studies), thus the term “causality” could be replaced by other less demanding terms such as “directionality” (which only suggests the existence of a time-sequence between X and Y). The term causality was used here because it is employed in most previously studies which applied similar analytical methods. When the time-resolution of the fcMRI did not allow the determination of the sequential order of brain activations, the term causality was replaced by “instantaneous dependence”, but when one center was clearly activated before the other center, the term used was “causation”, with the earlier activated nucleus being referred to as “causative nucleus” and the subsequent nucleus as “response nucleus”. The time resolution of present fMRI methods is 1.6 s, and only the activation sequences of centers performed within intervals of 1.6 s or 3.2 s (delayed causation) were considered here. The activation sequence cannot be interpreted as an excitation or direct activation carried out by the causative nucleus on the responsive nucleus. The activation sequence only means that during the execution of BG tasks these centers present a statistical trend to activate successively. With an example, the arrival of the night is usually followed by the turning on of the city lights, and this does not mean that the darkness can act directly on the light switches. This is particularly clear in the case of the delayed causations where the activation of a center is not followed by the activation of the other center until 3.2 s have elapsed. These slow transitions could be associated with the succession of tasks (e.g. the execution of a movement and the following adaptation of the muscle tone needed to balance the new body posture) rather than with the involvements of both centers in the same task.

Although present methods were designed to reject the most common spurious causations, artefactual interactions cannot be completely ruled out^11^. However, the methodological precautions used (e.g., long time-series, non-parametric significance tests, etc.) and the identification of the same functional interactions with different methods suggests that most functional relationships reported here are genuine. The use of different mathematical approaches provides clear advantages over other causal methods, including the possibility of identifying linear, non-linear, or more complex causations^11^. A limit of the present methodological approach is that it can identify individual interactions between two centers, but not multiple simultaneous interactions between the different centers of the same network. The independent component analysis^37–39^ and data-driven sparse GLM^40,41^ can simultaneously work with multiple regions but these multifactorial methods mainly identify linear interactions, functional relationships that do not always occur in the BG^31,42,43^ where many neurons display a non-linear dynamic^44–46^. Multifactorial methods have recently been developed that may work with multiple regions at the same time, but they do not provide an identification of BG interactions as exhaustive as the present method does (e.g., multiple correspondence analysis)^31^, and they do not identify causal relationships (multiple covariance method)^32^.

The joint application of present analytical methods offers additional advantages to their individual separate use. The most sensitive method for linear interactions is the PC. In this case, the spurious causations generated by the closed-loop arrangement of BGmC (which facilitates the repeated circulation of information) were prevented by using a partial correlation that subtracted the possible action of all the other centers of the network from the interaction between the two studied centers. The possible effect of performing many statistical contrasts was prevented by using a contrast method based on a block-shuffle permutation test. Non-linear interactions were studied with the GPDC, a method that avoids the possible interference of non-parametric noise and that can also identify linear relationships (although it is less sensitive than PC). Spurious causations were prevented here using partial correlations and the effect of repeating the statistical contrasts were avoided using shuffled data. Generally, PC and GPDC cannot identify complex relationships (e.g., those with phase transitions). The CMIknn method is the least sensitive and the most time-demanding method (a parallel version of the knn algorithm was used and several days were required for each time series, using 24 Intel Xeon Gold 6140 cores), but it can detect complex relationships that the other two methods cannot identify (e.g., those including nonlinear multiplicative noise). Considering the estimation of conditional mutual information, it scales approximately 0(n log(n)) regarding the time complexity, with n being the sample sizes, and linearly with the number of nuclei and the maximum time lag^11^. Therefore, the integrated application of these methods proved to be useful to identify functional BG interactions not observed with other methods, reducing the possibility of incorporating spurious causation into the BG model.

### The causality data and previous BG models

The M1 showed an instantaneous and a delayed dependency with the S1. The instantaneous dependence was observed during both the resting and motor tasks, and was analogous to that previously observed with other methods^31,47^. This permanent dependency could be at the basis of the continuous M1-S1 exchange of information that is necessary for checking the fit between the planned and executed movements^48,49^. The M1-S1 instantaneous dependence may be mediated by cortico-cortical pathways^50–52^, but the synchronous arrival of somatosensory information to the S1 and M1 could also be involved in this functional dependence^53–55^. The delayed M1 → S1 causation showed the M1 as the causative center of the S1 activity. The maintenance of body posture is normally performed by an active process that is continuously counterbalancing the unexpected fluctuations of the posture. The counterbalance process begins by identifying unexpected movements, a task that needs the comparison of the desired body posture (computed in the M1) and the actual posture (somatosensory data continuously transmitted from the thalamus to the S1). During the resting task, the delayed M1 → S1 causation could facilitate the M1 → S1 transmission of the body posture to be preserved, with the S1 activity being necessary to detect mismatches between expected and real data^56^. Finally, the M1-S1 instantaneous dependence could facilitate the continuous interchange of sensitive-motor information which is necessary for the fast correction of the body posture^57^.

Most functional relationships found between the M1 and BG were delayed (6 delayed vs. 2 instantaneous) task-related (7 TdC vs. 1 PC) dependencies. The M1 worked both as causative center of the GPe (during resting) and SN/Tal (during motion) activity, and as a response center for Tal activity (during resting). In addition, the M1 showed instantaneous dependency with the SN (during resting) and STN (during motion). Thus, most functional dependencies between the M1 and BG changed with the tasks, with the SN* → *M1 being the only causation observed during both the resting and motor tasks. The M1 is the exit door through which the cortico-subcortical motor loops of BG send information to α-motoneurons (for activating motor behaviors) and to ϒ-motoneurons (for modulating the posture and muscle tone). In the classical BG model, the M1 output is the result of a delicate balance between the excitatory (*M1 → ↑ Put → ↓ SN/GPi → ↑ Tal → ****↑**** M1*) and inhibitory (*M1 → ↑ Put → ↓ GPe → ↑ STN → ↑ SN/GPi → ↓ Tal → ↓ M1* and *M1 → ↑ STN → ↑ SN/GPi → ↓ Tal → ↓ M1*) action of BG on the M1, with the excitation-inhibition imbalance being the main cause of the motor disturbances in PD^16–24^. However, present data suggest that the M1 is much *more than a passive exit door* for the BG activity. The M1 showed an active influence on the behavior of many BG, and was particularly relevant for the GPe, STN, SN and Tal activity. The M1-BG interaction was markedly influenced by the motor task, suggesting that the M1 action on most BG is the result of a *task-dependent functional reconfiguration* of the BG loops. Many of the functional relationships of the M1 and BG were causative, with the M1 acting as the causative nucleus and BG centers as response nuclei. Taken together, these data suggest that the M1 has *executive functions* that organize the activity of BG according to the task to be performed, and not only as a passive exit door that the BG use to control the posture and the motor behavior. Contrary to that observed for the M1, the S1 showed few interactions with BG that were not modified by the motor task, which justifies the no inclusion of this area in most of the BGmC models^14–16,24^.

Although the Put receives massive excitatory inputs from the M1, no direct M1* → *Put causation was found here, neither during resting nor during motion. fMRI detects functional changes in brain centers only when a significant percentage of their neurons increase their activity and produce enough metabolic changes to generate the vasodilatation that the fMRI can identify. These facts probably do not occur in the Put because the medium-sized spiny cells, which are 95% of the neurons of this center, present a relatively low sensitivity to excitatory cortical afferences which is produced by the concurrent action of: (1) a hyperpolarized resting membrane potential, (2) a sustained inhibition generated by an inwardly rectifying potassium current, (3) a sustained collateral inhibition by the other GABA neurons of the putamen. Therefore, the response of these neurons needs the strong synchronous action of many cortical inputs and is generally local (only few striatal neurons at a time) and transient (persists as long as sufficient excitatory drive is present to maintain depolarization)^58–60^. Thus, the massive neuronal response necessary to induce the metabolic changes that the fMRI can detect are probably not very frequent in the Put.

The Put does not project to the M1 and its influence on this cortical area can be performed only through the direct and indirect pathways of the BGmC. These pathways present antagonistic actions on the brain cortex, the direct pathway activating and the indirect pathway inhibiting the M1 activity. This, and the fact that Put* → *M1 causation is probably lower than that induced by other cortical areas including the S1, may explain the finding of no Put* → *M1 statistical causation during the resting-task. The double-delayed Put* → M1* causation found during the motor-task suggests that the Put action on M1 activity is much more marked during the execution of voluntary movements than during resting. Although the Put* → M1* causation could be induced by an activation of the direct pathway or by an inhibition of the indirect pathway, the GPe data commented below is more in line with the second possibility.

The GPe is an intermediate center of the indirect pathway and is the only nucleus that is exclusively involved in this pathway in most BG models (GPi and SN are also involved in the direct pathway and STN in the hyperdirect pathway). Present data agree with this possibility, showing an instantaneous connectivity of the GPe with: (1) Put and STN (its preceding and subsequent centers in the indirect pathway); (2) GPi and SN (the two output centers of the indirect pathways); (3) Tal (a center that moves the GPi/SN activity to the M1). The finding of a permanent instantaneous dependence of the GPe with all the other BG suggests that the indirect pathway presents a basal influence on the BGmC that is not task-related. In addition to this permanent activity, the GPe worked as a response center for the SN, M1 and Put causative activity, a fact that was observed only during the resting intervals and which suggests that the indirect pathway is particularly relevant for the physiological functions that the BG perform during motor resting (e.g., maintaining posture, modulating muscle tone.). No GPe-M1 dependency was found during the motor activity, which suggests that the above commented Put* → M1* causation is linked to an inhibition of the indirect pathway more than to an activation of the direct pathway.

The STN showed an instantaneous dependency with most BG which agrees with its key role in both the indirect (M1-Put-GPe-STN-GPi/SN) and hyperdirect (M1-STN-GPi/SN) pathways. The STN showed a permanent functional dependency with the GPe (its input nucleus in the indirect pathway), and with the GPi, SN and Tal (its output centers in the indirect and hyperdirect pathways), data suggesting that the STN performs a permanent activity on the BGmC that does not change with the motor tasks. On the other hand, the STN showed an instantaneous dependence with M1 activity during motion that was not observed during resting, suggesting that the hyperdirect pathway is activated for the execution of voluntary movements^61^. The massive permanent interaction of the STN with most BG and the activation of the STN-M1 relationship observed during motion agree with the therapeutic action induced by the modification of the STN activity on both the passive (hypertonia.) and the active (bradykinesia, hypokinesia..) motor disorders of PD^62^.

The classical BG model suggests that the SN and GPi are involved in the integration of the activity of the direct, indirect and hyperdirect pathways, with both centers performing similar functions in the BGmC where they act as a single functional entity^16,18^. Present data do not support this possibility. Although the SN and GPi showed a permanent dependency between them and both centers showed permanent interactions with the GPe and STN (which suggests the SN and GPi perform similar functions), the permanent dependence of the GPi with the Put was not found for the SN and the permanent dependence of the SN with S1 was not found for the GPi (which suggests that the SN and GPi perform different functions). In addition, the SN-Tal and SN-M1 relationship observed during resting was not found for the GPi, and the M1 → SN causation observed during motion was not found for the GPi. Thus, the differences found for the functional connectivity of the SN and GPi with the other BG indicate that they perform different functions in the GBmC, particularly during the execution of voluntary movements. However, we must be cautious with this interpretation of data since the SN in this study includes both its compacta and reticulate sub-regions. These sub-regions present a different connectivity and are probably involved in different functions and, although they can be clearly segregated with anatomical^63^ and electrophysiological^64^ methods in animals, their MRI segregation in humans may provide not reliable fMRI data. Thus, the segregated analysis of both areas was avoided here.

The Tal showed a permanent dependence with the Put, GPe, STN and S1. The Put-GPe interaction could be mediated by the thalamo-striatal glutamatergic projections, which acting on the medium-sized spiny neurons of the indirect pathway could be at the basis of the Tal-GPe and Tal-STN permanent dependence. The Tal-S1 permanent dependence could be caused by the fast feed-back interactions observed between the Tal and S1 neurons (supported by Tal* → S1* and S1* → *Tal glutamatergic projections). The role of this feed-back circuit within the BG motor-loop has not been elucidated but, bearing in mind the role proposed for the same circuit in other cortex-BG loops^65^, it could be at the basis of the motor attention which is often activated when the body posture or the progression of motor actions do not follow the initial plans.

The previous comments are an attempt to look at the data presented here from the perspective of the classical BG model. The classical model uses the firing-rate of neurons and their fast excitatory/inhibitory interactions recorded in the brain of experimental animals to estimate the activity of each BG and the global dynamics of the BG networks in the human brain. The present study uses the metabolic dynamics of the human BG obtained during the execution of different tasks to estimate the causation relationships between BG and their re-configuration during the execution of different motor tasks. The nature of the data (electrophysiological vs. metabolic), and the spatial (microns vs. millimeters) and time (milliseconds vs. seconds) scenarios of both approaches are different, and the models constructed from these different approaches should be considered as complementary models and not as incompatible models.

### The activity of the human BG according to the causation data: a global view

**Figure 4:**
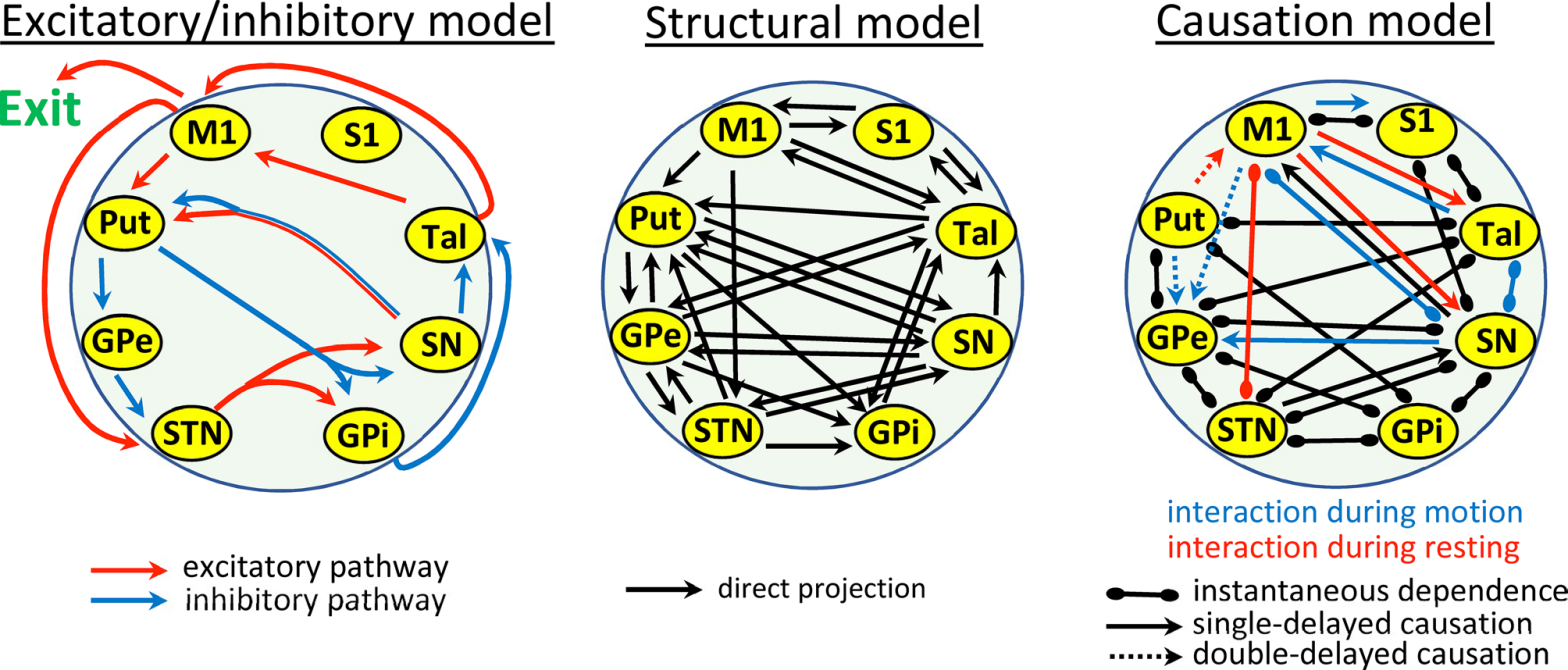
Basal ganglia models. The classical basal ganglia model (left-side) is mainly based on the excitatory (red) and inhibitory (blue) interactions of the BG centers involved in the direct, indirect and hyperdirect pathways of the cortico-subcortical loops. The structural model shows most of the BG pathways, also including those involved in subcortical loops. The causation model (right-side) includes all the instantaneous (lines) and delayed (arrows) interactions observed during resting (blue), motion (red) and both resting + motion (black). M1: primary motor cortex, S1: primary somato-sensory cortex, Put: putamen, GPe: external globus pallidum, STN: subthalamic nucleus, GPi: internal globus pallidum, SN: substantia nigra, Tal: motor thalamus.

In the classical model, the goal of the excitatory/inhibitory interactions of the successive nuclei of the BGmC is the modulation of the M1 activity (left-side Fig. 4), with the PD disorders being induced by the low activity of the M1 induced by the preponderance of the indirect pathway (↓dopamine → ↑Put → ↓GPe → ↑STN → ↑GPi/SNr → ↓Tal → ↓M1) over the direct pathway (↓dopamine → ↓Put → ↑SNr/GPi → ↓Tal → ↓M1). Present data suggest a more complex organization of BG where each nucleus may directly or indirectly interact with any of the other BG. The reentrant signaling induced by feed-back loops has been proposed as a mechanism that facilitates the functional coupling of many cortical areas^66^. The circular arrangement of the cortico-subcortical BG loops facilitates the reentrant signaling and may be at the basis of many of the functional interactions observed here, even in the case of nuclei which do not present direct anatomical pathways between them. For instance, the M1 showed a complex influence on the activity of most BG even when it only projects to the Put and STN and receives direct input from the Tal.

The classical model uses stable functional excitatory/inhibitory interactions of BG nuclei to explain the BG functions. The present data suggest that the functional interactions of BG may be quickly reconfigured according to the task that is being executed. For instance, when the task is to maintain a stable body posture, the M1 coordinates its activity with the SN and modulates the activity of the GPe. However, when the task is to perform voluntary movements, the M1 coordinates its activity with the STN and modulates the activity of the SN and Tal. The feed-back return of BG to the M1 will be different in both cases, and thus the action of the M1 on the body posture and motor activity may also be different.

The interaction of the direct, indirect and hyperdirect cortico-subcortical circuits are used in the classical model to explain the motor functions of the BGmC. However, there are a number of subcortical loops which may facilitate the back-propagation of the information flux, the cross transmission between BG nuclei (not following the BG-loop) and the sub-cortical reentry of information, and which have been comparatively little studied. One of the characteristics that have driven the use of the classical BG model is its “simplicity”, a feature that is lost when all the BG pathways are included in the functional model (structural model in Fig. 4). Present methods do not provide evidence about the path followed by the information within the BG. However, they were useful here to identify the functional connections of BG nuclei, even though the pathways that support this functional connectivity could not be identified (right-side Fig. 4). The present data also showed the nuclei that are commanding the inter-nuclei interactions (causative nucleus). The causative nucleus could not be identified in all cases, but this is the consequence of the limited time-resolution of the fMRI and not of the analytical method applied. The instantaneous dependency does not mean the non-existence of a causative nucleus, it may simply be an indicator that the time resolution of the BOLD signal is not enough to detect the origin of fast functional interactions, a limitation that in the future could be solved with the introduction of faster fMRI techniques.

There is previous evidence showing the functional arrangement of more than two BG during the execution of particular tasks^31,32^. Present methods can provide an exhaustive list of all interactions that have occurred between two nuclei of the BG, but they cannot identify the possible functional grouping of three or more of the BG nuclei. This is a limitation of the analytical method and could benefit from the development of new mathematical approaches that can identify causation in more than two areas at once. Finally, the nuclei of the BGMC present a number of input/output direct and indirect relationships with many cortical (e.g. supplementary motor area, pre-suplementary motor area, dorsal–ventral and rostral-caudal parts of the premotor cortex, rostral and caudal parts of the cingulate motor areas, precuneus, different areas of the prefrontal cortex..) and subcortical (e.g. pedunculo pontine nucleus, amygdala, red nucleus of the stria terminals, nucleus accumbens, superior colliculus, periacueductal grey, parabrachial nucleus..) areas which probably determinates its short and long-term dynamic. The study of the functional interactions between these centers and the BGmC nuclei will be necessary to gain a deeper understanding of the BG physiology, and of the biological basis of motor disorder in PD and other neurological illnesses. The use of causation methods can help the progress of these studies.

## Methods

### Participants

Twenty right-handed volunteers with no history of neurological or mental disease participated in this study (10 males and 10 females between 23–65 years of age; 45.4 ± 9.1 years old). Written informed consent was provided by all participants, all procedures were in accordance with the ethical standard of the 1964 Helsinki declaration, and the study was approved by an institutional review board (Institutional Human Studies Committee of La Laguna University).

### Data collection

The basic experimental procedures were similar to those reported in recent studies^67,68^, but using two experimental conditions, motor-resting (subjects maintained their body posture and did not perform any planned movement) and motor-task (subjects performed a repetitive sequence of finger extensions/flexions with the right-hand). BOLD-contrast images (4 × 4x4 mm voxels in-plane resolution; echo-planar imaging with repetition time 1.6 s; echo time 21.6 ms; flip angle 90º) were recorded in blocks of 100 volumes in the following sequence: motor block → resting block → motor block → resting block (400 total volumes/subject = 100 volumes × 2 motor-blocks × 2 resting-blocks). fMRI data were co-registered with 3D anatomical images (1 × 1 × 1 mm voxel resolution; repetition time 7.6 ms; echo time 1.6 ms; flip angle 12°; 250 × 250 mm field of view; 256 × 256 sampling matrix). A representative region of interest (ROI) of each BG was located on a subject-by-subject basis by considering: (1) the Talairach coordinates, (2) the shape of the nucleus, and (3) the anatomical relationship of the nucleus with neighboring structures. All regions were identified in coronal slices located 4–27 mm posterior to the anterior commissure and according to a previously reported procedure^68^. All data sets were normalized to the Talairach space (Table 3 shows the position and size of ROIs).

**Table 3:** Coordinates (Talairach) are shown in mm.

	X	Y	Z	Size
Primary somatosensory cortex	31.1 ± 3.9	− 20.1 ± 5.1	48.3 ± 4.62	34.8 ± 7.1
Primary motor cortex	38.2 ± 4.6	− 17.2 ± 4.8	45.1 ± 6.3	35.4 ± 12.0
Putamen	24.0 ± 0.8	− 4.9 ± 1.0	0.2 ± 0.3	22.3 ± 3.4
External pallidum	12.2 ± 4.3	− 2.1 ± 0.7	2.6 ± 1.1	9.6 ± 2.1
Internal pallidum	13.2 ± 1.4	− 5.8 ± 1.3	− 1.4 ± 1.0	11.2 ± 1.1
Subthalamic nucleus	9.7 ± 1.4	− 12.1 ± 2.1	− 4.2 ± 2.7	2.8 ± 0.5
Substantia nigra	7.1 ± 1.1	− 17.7 ± 1.4	− 7.6 ± 1.9	45.6 ± 7.8
Ventral-anterior thalamus	8.6 ± 1.4	− 9.1 ± 1.1	6.8 ± 2.1	24.4 ± 7.8

The size of the ROIs is shown by the number of their voxels.

### Data preprocessing

The data preprocessing included a slice scan time correction, a 3D motion correction, and a time filter which eliminates frequencies below 0.009 Hz. Studies with images showing a displacement > 0.5 mm or a rotation > 0.5degrees were removed. No spatial smoothing was performed. Residual motion artifacts and physiological signals unrelated to neural activity (e.g., respiration, cardiac activity) were removed by regressing the BOLD signals recorded throughout the brain with the mean average of the BOLD signals recorded in white matter and brain ventricles^69,70^.

For each brain nucleus, the time series for all the participants were concatenated to obtain two data sequences, one for the motor case and the other for the resting case. As a first step, data for each subject were normalized around the mean. Then each 100 samples block (motor or resting) was concatenated with the other blocks of the same type, two for each person, for the whole set of participants, obtaining a single time series of 100 volumes × 2 motor/resting blocks × 20 participants = 4000 samples. In order to avoid spurious correlations between series due to block concatenation, the first and last 5 samples of each block were filtered using a gaussian moving average window of size 5, smoothing the transitions between different recordings.

The time series for each nucleus and of the same type (motor/resting), Xi, were joined to create multivariate time series, X, of dimension N (with N being the number of brain nuclei considered; N = 8). For each time step Xt = (X1t, X2t,…,XNt).

### Causality analysis

In this work, a causal network algorithm has been used to infer dependencies between the eight BG nuclei. In particular, the PCMCI method was applied to the multivariate time series for the motor and resting cases^71^. This causal discovery method consists of two steps. In the first one, it uses a version of the algorithm proposed by Peter and Clark (PC)^72^ but only to select the conditions necessary for the next step, reconstructing the causal parents of each nucleus through iterative conditional independence tests. In the second one, the momentary conditional independence (MCI) test is applied, which uses the sets of parents to determine the strength of causal relationships. Specifically, Python package Time Series Graph Based Measures of Information Transfer (TiGraMITe), available at https://github.com/jakobrunge/tigramite.git, has been used.

To find causal relationships between the different nuclei, at different time lags, it is necessary to identify processes that directly influence each of them, in other words, their parents. To estimate the relationship between two processes (nuclei signals), X^i^ and X^j^, a particular definition of conditional independence is used. In general, conditional independence of X^i^_t − λ_ and X^j^_t_ given Z, denoted by X^i^_t − λ_ ∐ X^j^_t_ | Z, can be expressed in terms of the corresponding conditional probabilities:

$$ {\text{X}}^{{\text{i}}} _{{{\text{t}}\, - \,\lambda }} \coprod {\text{X}}^{{\text{j}}} _{{\text{t}}} |{\text{ Z}} \Leftrightarrow {\text{p}}\left( {{\text{x}}^{{\text{i}}} _{{{\text{t}}\, - \,\lambda }} ,{\text{x}}^{{\text{j}}} _{{\text{t}}} |{\text{z}}} \right)\, = \,{\text{p}}\left( {{\text{x}}^{{\text{i}}} _{{{\text{t}} - \lambda }} \left| {{\text{z}}){\text{p}}({\text{x}}^{{\text{j}}} _{{\text{t}}} } \right|{\text{z}}} \right)\forall {\text{x}}^{{\text{i}}} _{{{\text{t}} - \lambda }} ,{\text{x}}^{{\text{j}}} _{{\text{t}}} ,{\text{ z}} $$

where X^i^_t−τ_ indicates the time series corresponding to nucleus i at lag τ, and Z is a subset of all other processes that potentially influence the relationship between the two processes being tested, that is, a subset of {X^1^_t_, X^2^_t_, …, X^N^_t_, X^1^_t−1_, X^2^_t−1,_ …, X^N^_t−1_,…,X^1^_t−T_, X^2^_t−T,_…,X^N^_t−T_}, where T is the maximum lag considered.

TiGraMITe provides several test statistics to test independence hypotheses, which are typically based on specific assumptions about the underlying dependence between processes, three of which have been used in this study. The first one assumes linear relationships between variables. In this case, the conditional independence can be tested by removing the linear influence of Z from both X^i^_t−λ_ and X^j^_t_ and testing for the correlation between their residuals, that is, computing the corresponding partial correlation (PC), ⍴(X^i^_t−λ_, X^j^_t_ | Z). The second one uses a non-parametric regression based on gaussian process regression and a distance correlation (GPDC)^35^ to test the dependence, allowing the detection of those dependences that are non-linear. The last one is the conditional mutual information test based on nearest-neighbor (CMIknn) estimator^36^. It is the most general dependency measure, and makes no assumptions about the parametric form of the dependencies by directly estimating the underlying joint density. The linear method is based on classical statistics and provides a robust theoretical background. The non-parametric and model-free methods allow the detection of non-linear relationships in complex systems, but they are based on weaker theoretical results^11^. In all cases, even in the linear one, the statistical significance of conditional independence tests were computed using a block-shuffle permutation test^73^. This prevents the assumption that the samples are independent and identically distributed, as required by analytic methods, because the time series are usually autocorrelated. In this study, a two-sided significance level of 0.01 and a maximum time lag of T = 2 (3.2 s) were chosen, implying that parent processes occurred before this time or those with a significance below 99% are neglected.

Although causal discovery algorithms are a powerful tool for studying dependencies between variables, they are based, like all statistical methods, on different assumptions than cannot always be fulfilled in brain studies. In this case, the most important assumptions are time-order, causal sufficiency, the causal Markov condition, and faithfulness^11^. Time-order means that causes precede effects. Causal sufficiency assumes that all direct common drivers are in the set of observed time series, in other words, there are no other unobserved processes that directly or indirectly influence any other pair of the studied processes. Causal Markov condition implies that once X^i^_t_ parent values are known, all other variables in the past are not relevant for predicting the value of X^i^_t_. Faithfulness, together with causal Markov condition, guarantees that a measured statistical dependence is due to some, direct or indirect, causal mechanism and, conversely, a measured independence implies that there is no direct causal mechanism.

Despite the limitations due to the above assumptions, the method of reconstructing causal networks from time series has multiple advantages over simpler and commonly used methods, such as those based on the study of correlations between pairs of time series. Correlations do not allow us to infer the direction of the relationship, so they are not suitable for studying causal effects. Correlations between the time series of two nuclei can also be highly sensitive to possible indirect connections through a third nucleus. In fact, in complex systems, there are generally many more indirect than direct connections, and a bivariate analysis would show many statistically significant correlations, but it cannot establish causal relationships between the different nuclei. Moreover, if there is a common driver, that is, one nucleus exerts a causal action on two others, these, in turn, will show some correlation, even if there is no direct interaction between them. The causal discovery algorithms overcome all these spurious relationships, at least among the observed variables included in the analysis^74^.

### Compliance with ethical standards

Experiments were conducted in in agreement with European Union directives (Ley 32/2007, Real Decreto 1201/2005, Ley 9/2003 and Real Decreto 178/2004, 86/609/CCE, 91/628/CEE and 92/65/CEE), and was supported by a Local Institutional Human Studies Committee. This article does not contain any studies with animals performed by any of the authors. Informed consent was obtained from all individual participants included in the study.

## Data Availability

All data generated or analyzed during this study are included in this manuscript and are available on request.
